# Resting-state fMRI in sleeping infants more closely resembles adult sleep than adult wakefulness

**DOI:** 10.1371/journal.pone.0188122

**Published:** 2017-11-17

**Authors:** Anish Mitra, Abraham Z. Snyder, Enzo Tagliazucchi, Helmut Laufs, Jed Elison, Robert W. Emerson, Mark D. Shen, Jason J. Wolff, Kelly N. Botteron, Stephen Dager, Annette M. Estes, Alan Evans, Guido Gerig, Heather C. Hazlett, Sarah J. Paterson, Robert T. Schultz, Martin A. Styner, Lonnie Zwaigenbaum, The IBIS Network, Bradley L. Schlaggar, Joseph Piven, John R. Pruett, Marcus Raichle

**Affiliations:** 1 Department of Radiology, Washington University School of Medicine, Saint Louis, Missouri, United States of America; 2 Department of Neurology, Washington University School of Medicine, Saint Louis, Missouri, United States of America; 3 Departamento de Fisica, Universidad de Buenos Aires, Buenos Aires, Argentina; 4 Department of Neurology, Christian-Albrechts-University Kiel, Germany; 5 Institute of Child Development, University of Minnesota, Minneapolis, Minnesota, United States of America; 6 Department of Psychiatry, University of North Carolina, Chapel Hill, North Carolina, United States of America; 7 Department of Radiology, University of Washington, Seattle, Washington, United States of America; 8 Department of Psychology, University of Washington, Seattle, Washington, United States of America; 9 Montreal Neurological Institute, McGill University, Montreal, Quebec, Canada; 10 Department of Psychiatry, New York University, New York, New York, United States of America; 11 Department of Pediatrics, University of Pennsylvania, Philadelphia, Pennsylvania, United States of America; 12 Department of Pediatrics, University of Alberta, Edmonton, Alberta, Canada; 13 Department of Psychiatry, Washington University School of Medicine, Saint Louis, Missouri, United States of America; University of Texas at Austin, UNITED STATES

## Abstract

Resting state functional magnetic resonance imaging (rs-fMRI) in infants enables important studies of functional brain organization early in human development. However, rs-fMRI in infants has universally been obtained during sleep to reduce participant motion artifact, raising the question of whether differences in functional organization between awake adults and sleeping infants that are commonly attributed to development may instead derive, at least in part, from sleep. This question is especially important as rs-fMRI differences in adult wake vs. sleep are well documented. To investigate this question, we compared functional connectivity and BOLD signal propagation patterns in 6, 12, and 24 month old sleeping infants with patterns in adult wakefulness and non-REM sleep. We find that important functional connectivity features seen during infant sleep closely resemble those seen during adult sleep, including reduced default mode network functional connectivity. However, we also find differences between infant and adult sleep, especially in thalamic BOLD signal propagation patterns. These findings highlight the importance of considering sleep state when drawing developmental inferences in infant rs-fMRI.

## Introduction

Resting-state functional magnetic resonance imaging (rs-fMRI) in human infants has made it possible to detect key features of early brain functional organization, including the existence of resting state networks at early ages [1, 2], developmental changes [3, 4], and even neural correlates of prematurity [5]. However, infant neuroimaging generally requires the subjects to be asleep in the scanner ([1, 6]; early studies also used sedation in place of natural sleep [7]). Otherwise, motion inside the scanner would make it extremely challenging to acquire interpretable images.

The fact that rs-fMRI in infants is acquired during sleep introduces a potential interpretational confound when comparing these data to rs-fMRI collected from awake adults. For example, it has been reported that the infant default mode network (DMN) exhibits weaker functional connectivity than in adults [8], but it has also been shown by a number of independent groups that sleep itself reduces functional connectivity in the adult DMN [9–13]. These findings raise the question of whether reduced DMN functional connectivity in infants is attributable to development, sleep, or a combination of both. More broadly, it is not understood whether the functional organization of rs-fMRI in sleeping infants more closely resembles that of awake adults or sleeping adults.

Ideally, the separate effects of sleep vs. wake, and even sleep stage, on infant rs-fMRI analyses could be parsed out by imaging babies using combined EEG/fMRI during sleep and wakefulness. Unfortunately, to the best of our knowledge, the technical challenges inherent both to imaging awake babies and to acquiring combined EEG/fMRI in babies have not been solved. Lacking combined EEG/fMRI data in awake and asleep children, we can instead indirectly infer the effects of sleep on early childhood rs-fMRI analyses by comparing rs-fMRI in sleeping children to combined EEG/fMRI in young, healthy adults obtained in wakefulness and non-rapid eye moment (NREM) sleep. Specifically, investigating whether the organization of rs-fMRI in sleeping infants and toddlers more closely resembles awake or asleep adults informs the question of whether some effects previously attributable to development may instead more likely result from wake vs. sleep effects.

To address this question, we compare the functional organization of rs-fMRI in sleeping 6, 12, and 24 month children vs. rs-fMRI acquired in young, healthy adults during both wakefulness and non-rapid eye moment (NREM) sleep. Thus, our study is explicitly developmental in design. Analytically, we examine two features of the functional organization of the resting-state brain using fMRI BOLD imaging: functional connectivity (zero-lag covariance) and propagation (temporal lag structure) of the BOLD signal ([14, 15]; reviewed in [16]). Functional connectivity (FC) is a common analysis strategy for identifying spatially distinct topographies of correlated activity in the brain [17]. Whereas FC identifies spatially segregated networks (also called resting state networks), we have recently shown that temporal lags in the BOLD signal reveal propagation of activity within and across networks, a signature of infra-slow neural communication [16]. Importantly, the propagation of spontaneous BOLD signals is markedly altered across adult wake vs. sleep [10, 18], providing an analytic tool for differentiating the organization of rs-fMRI in wake vs. sleep.

## Methods

### Subjects

There are two cohorts of subjects in this study, young adults and children (ages 6, 12, and 24 months), data from both of which have been previously published. For the young adults, written informed consent was obtained from all subjects whose data was analyzed in this study, and data collection for this study was approved by the Goethe University ethics committee. Childhood data was acquired from an NIH-funded Autism Centers of Excellence (ACE) network study, referred to as the ‘Infant Brain Imaging Study’ (IBIS). The network includes four clinical data collection sites (University of North Carolina at Chapel Hill, University of Washington, Children’s Hospital of Philadelphia, Washington University in St. Louis), a Data Coordinating Center at the Montreal Neurological Institute (McGill University), and two image processing sites (University of Utah and UNC). Data collection sites had study protocols approval from their Institutional Review Boards (IRB), and all enrolled subjects had informed consent provided by parent/guardian. Written informed consent was also obtained from all adult subjects whose data was analyzed in this study, and data collection for this study was approved by the Goethe University ethics committee.

### Adult subjects

63 non-sleep-deprived young adult subjects were scanned in the evening (starting at ∼8:00 PM). Subjects were instructed to keep their eyes closed during wakefulness and were allowed to sleep. Hypnograms were inspected to identify epochs of contiguous sleep stages lasting at least 5 min (150 volumes). These criteria yielded 39 subjects contributing to the present analyses. Included are 70 epochs of wakefulness, 52 epochs of N1 sleep, 47 epochs of N2 sleep, and 38 epochs of N3 sleep (SWS). Detailed sleep architectures and demographic details of each participant have been previously published [19], and are summarized in Table 1.

**Table 1:** fMRI acquisition/registration parameters in pediatric and adult data.

	Pediatric subjects	Adult subjects
Citation	Pruett et al., 2015	Tagliazucchi et al., 2012
Age (yr)	0.53 ± 0.04, 1.04 ± 0.04, 2.06 ± 0.04	24 ± 5
Scanner	3T Siemens TIM Trio	3T Siemens TIM Trio
Acquisition resolution (mm)	4 × 4 × 4	3 × 3 × 4
Repetition time (sec), TE (msec)	2.5, 27	2.08, 30
Frames × fMRI runs	200 × 2 (minimum)	1505 × 1
EPI atlas registration scheme	EPI→T2W→T2W-atlas	EPI→T1W→T1W-atlas
Mean, standard deviation DVARS	5.0 ± 0.96, 4.96 ± 0.99, 4.99 ± 1.01	5.02 ± 1.03

### Pediatric subjects

In a broader study of autism spectrum disorders (ASD), high-familial-risk-for-ASD and low-familial-risk-for-ASD infant cohorts (where risk was defined by an older sibling who either was or was not diagnosed with ASD) were recruited as part of a National Institutes of Health-funded, multi-site, Autism Centers of Excellence (ACE) Network study: the Infant Brain Imaging Study (IBIS). Subjects were excluded for comorbid medical or neurological diagnoses influencing growth, development, or cognition; prior genetic conditions; premature birth or low birth weight; maternal substance abuse during pregnancy; contraindication for MRI; or familial history of psychosis, schizophrenia, or bipolar disorder [3, 20]. Only low-risk infants were included in the present analysis. Low-risk was defined as having at least one typically developing older sibling and no first- or second-degree family members with ASD or intellectual disability. This paper includes data only from low-familial risk infants who, at 24 months of age, did not meet criteria for ASD according to clinical best estimate using DSM-IV-TR criteria applied to all available information. This evaluation was based on a comprehensive battery of behavioral assessments including the Autism Diagnostic Observation Schedule (ADOS: [21]). ADOS and all other testing and interview data were independently reviewed by expert clinicians for DSM-IV-TR criteria for autistic disorder or pervasive developmental disorder not otherwise specified.

Children were scanned at ages 6, 12, and 24 months. Three groups were defined: 6 month olds (17 subjects), 12 month olds (17 subjects), and 24 month olds (11 subjects). Thus, the total number of pediatric subjects was 45. These data sets represent the highest quality subset, as determined using the DVARS measure [22, 23], of 46 data sets collected at 6 months, 51 data sets collected at 12 months, and 44 data sets collected at 24 months (see S1 Fig for details). Not all child participants were scanned at all ages, and longitudinally studied individuals did not always yield high-quality data at all ages. Consequently, this analysis is purely cross-sectional (no participant appears in multiple age categories). Additional administrative details have been previously published [3] and are summarized in Table 1.

### Adult EEG–fMRI acquisition and sleep stage determination

Acquisition parameters and details for these data have been previously published [19]. fMRI was acquired using a 3 T scanner (Siemens Trio) with optimized polysomnographic settings (1,505 volumes of T2*-weighted echo planar images, repetition time/echo time = 2,080 ms/30 ms, matrix = 64 × 64, voxel size = 3 × 3 × 2 mm^3^, distance factor = 50%; field of view = 192 mm^2^). 30 EEG channels were simultaneously recorded using a modified cap (EASYCAP) with FCz as reference (sampling rate = 5 kHz, low pass filter = 250 Hz, high pass filter = 0.016 Hz). MRI and pulse artifact correction were performed based on the average artifact subtraction method [24] as implemented in Vision Analyzer2 (Brain Products) followed by ICA-based rejection of residual artifact components (CBC parameters; Vision Analyzer). EEG sleep staging (N0 = wakefulness, N1-N3 = NREM sleep) was done by an expert according to the American Academy of Sleep Medicine (AASM) criteria [25], as previously published [19].

### Infant fMRI acquisition

All scans were acquired at the Infant Brain Imaging Study (IBIS) Network clinical sites using identical 3-T Siemens TIM Trio scanners (Siemens Medical Solutions, Malvern, PA) equipped with standard 12-channel head coils. All imaging was performed while subjects were naturally sleeping. Techniques that enhance the success of this approach include (1) scanning at a time of day that coincides with the infant's usual sleep schedule; (2) pre-conditioning at home for several days by exposure to pre-recoded scanner noise during natural sleep (3) swaddling; (4) placing sound attenuating ear muffs on the infant during scanning; (5) stationing a parent beside the infant in the magnet room. The IBIS imaging protocol includes T1-weighted (T1W) and T2W anatomical imaging, 25-direction DTI and 65-direction HARDI DWI diffusion sequences, and resting state fMRI. This study made use of the 3-D sagittal T2W sequence (TE = 497 ms, TR = 3200 ms, matrix 256 × 256 × 160, 1 mm^3^ voxels). Functional images were collected as a gradient-echo echo planar image (EPI) (TE = 27 ms, TR = 2500 ms, voxel size 4 mm × 4 mm × 4 mm, flip angle 90°, field of view 256 mm, matrix 64 × 64, bandwidth 1906 Hz). All presently analyzed infants provided at least two fMRI runs with a minimum of 200 total frames of data (8.3 min), including at least 4 continuous (uncensored) 60-second blocks.

### Preprocessing of adult fMRI data

The presently used adult fMRI preprocessing procedures have been extensively described [10, 15]. Initial preprocessing included compensation for slice-dependent time shifts, elimination of systematic odd-even slice intensity differences due to interleaved acquisition, and rigid body correction of head movement within and across runs [26]. The fMRI data were intensity scaled (one multiplicative constant over all voxels and frames) to obtain a whole brain mode value of 1000 [27]. Such scaling facilitates the computation of variance measures for purposes of quality assessment but does not alter computed correlations. Atlas registration was computed by registration of individual T1-weighted (T1W) images to a 711-2B space template image [26]. The 711-2B template represents Talairach space much as does the MNI152 template but it is about 5% smaller in linear dimensions. Atlas transformation was achieved by composition of affine transforms connecting the fMRI volumes with the structural images (fMRI average volume→T2W→T1W→template). The volumetric timeseries were resampled in (3mm)^3^ atlas space including head movement correction and atlas transformation in a single resampling step.

Additional preprocessing in preparation for functional connectivity and latency analyses included spatial smoothing (6 mm full width at half maximum (FWHM) Gaussian blur in each direction), voxel-wise removal of linear trends over each fMRI run and temporal low-pass filtering retaining frequencies below 0.1 Hz. Spurious variance was reduced by regression of nuisance waveforms derived from 6 retrospective head motion parameters (3 translation + 3 rotation, no derivatives) and timeseries extracted from regions (of “non-interest”) in white matter and ventricles defined in atlas space. Motion regressors were filtered identically to the fMRI data to prevent introduction of artifact generated by spectral mismatch [28]. The global signal (fMRI timeseries averaged over the brain) and its first derivative were included as nuisance regressors [29, 30]. Following nuisance regression, frames (volumes) prominently affected by head motion were identified using the DVARS (differentiated rms variance) measure [22, 23]. The censoring criterion applied to the adult data was > 0.5% frame-to-frame rms signal change. Censored frames were subsequently excluded from all resting state functional connectivity and lag analyses. Frame-censoring statistics are reported in Table 1.

### Preprocessing of pediatric fMRI data

Preprocessing of the pediatric fMRI data followed previously described procedures [3, 23]. As these procedures are largely parallel to the above-described adult case, only pertinent differences are detailed here. T1-weighted gray/white contrast is poorly developed in infants. Hence, atlas registration was computed via T2-weighted structural images. Age specific (6-, 12-, 24-month) T2-weighted atlas-representative templates representing 711-2B space were created based on the atlases generated by Fonov and colleagues [31]. Infant fMRI is much more distorted by magnetization inhomogeneities than is adult fMRI. Accordingly, distortion correction was computed using the prelude module in fsl [32]. Magnetization inhomogeneity field maps were either measured (data acquired mostly after 2012) or, when this measurement was not available (data acquired mostly before 2012), approximated using the method of Gholipour [33]. Atlas transformation of the fMRI data was computed by transform composition (fMRI average volume→T2W→template). The volumetric time series then were resampled in atlas space ((3mm) ^3^ voxels) including correction for head movement and EPI distortions in a single resampling step. Additional preprocessing in preparation for functional connectivity and latency analyses was largely as in the adult case except that the frame censoring criterion was 0.9% frame-to-frame rms intensity change evaluated in spatially smoothed data (10mm FWHM blur internal to the DVARS module). Frame censoring statistics are reported in Table 1.

### ROI definition

All present analyses were conducted by extracting preprocessed, fMRI time series from 6526 ROIs defined as (6mm)^3^ cubes restricted to gray matter in 711-2B atlas space. For details of the gray mask definition, please see [10, 15]. In the following text, we refer to the (6mm)^3^ cubes simply as voxels.

### Functional connectivity

We use the standard covariance formula for computing functional connectivity between pairs of time series. Thus, for a pair of time series, *x*_*i*_ and *x*_*j*_, zero-lag functional connectivity is computed as a sum over frames,

$$C_{x_{i}x_{j}}=\frac{1}{m}\sum_{k}x_{i,k}∙x_{j,k},$$ [E1]

where *i* and *j* index ROI, *k* indexes frame (volume) and *m* is total number of frames in an fMRI run (excluding censored frames). Thus, *C*_*xixj*_ is a matrix. We display functional connectivity matrices with voxels sorted into networks as defined in [34] and include only voxels with a 95% likelihood of single network affiliation. This selective display is for illustrative purposes only; all analyses are computed over all voxels in gray matter.

### Computation of lag between BOLD time series

Our method for computing lags between time series has been previously published [15]. In brief, we generalize the assumption of exact temporal synchrony and compute lagged cross-covariance functions. Thus,

$$C_{x_{i}x_{j}}(\Delta )=\frac{1}{n}\sum_{k}x_{i,k}∙x_{j,k+\Delta },$$ [E2a]

where Δ is a temporal displacement in units of frames. Thus, *C*_*xixj*_(0) is conventional, zero-lag FC. N.B.: Since all time series are made zero mean during preprocessing, the factors in [E2a] are relative to zero. Using parabolic interpolation, the temporal displacement at which *C*_*xixj*_ is maximal can be determined at a temporal resolution much finer than the frame TR [15]. Accordingly, for purposes of lag estimation, we can express *C*_*xixj*_(Δ) as the integral,

$$C_{x_{i}x_{j}}\left(\tau \right)=\frac{1}{T}\int x_{i}\left(t+\tau \right)∙x_{j}\left(t\right)dt,$$ [E2b]

where *τ* is the lag in units of time (generally a fraction of TR) and *T* is total length of data included in the integral. The value of *τ* at which *C*_*xixj*_(τ) exhibits an extremum defines the temporal lag (equivalently, delay) between signals *x*_*i*_ and *x*_*j*_. Additional discussion of parabolic interpolation is given in the supplemental material of [18].

Given a set of *n* time series, {*x*_1_(*t*),*x*_2_(*t*),…,*x*_*n*_(*t*)}, finding all *τ*_*i*,*j*_ corresponding to the extrema of *C*_*xixj*_(*τ*) yields the anti-symmetric, time delay matrix,

$$TD=\left[\begin{array}{ccc} \tau_{1,1} & \cdots & \tau_{1,n}\\ \vdots & \ddots & \vdots \\ {-\tau }_{1,n} & \cdots & \tau_{n,n} \end{array}\right].$$ [E3]

The diagonal entries of *TD* are necessarily zero, as any time series has zero lag with itself. Moreover, *τ*_*i*,*j*_ = −τ_*j*,*I*_, since time series *x*_*i*_(*t*) preceding *x*_*j*_(*t*) implies that *x*_*j*_(*t*) follows *x*_*i*_(*t*) by the same interval. Here, the timeseries are extracted from (6mm)^3^ cubic voxels evenly distributed throughout gray matter in the whole brain [15]. Seed-based lag maps are computed as described above, but with reference to an average timecourse derived from a region of interest.

We project the multivariate data represented in the *TD* matrix onto one-dimensional maps using the technique described by Nikolic and colleagues [35, 36]. We refer to these one-dimensional maps as lag projections. Operationally, the projection is done by taking the mean across the columns of *TD* [E3], that is,

$$T_{p}=[\sum_{j=1}^{n}\tau_{1,j}\ldots \sum_{j=1}^{n}\tau_{n,j}].$$ [E4]

The column-wise average (lag projection) reflects the extent to which a given voxel is early or late with respect to all the other voxels in the brain [15]. We refer to displays of these values as lag projection maps.

Group level covariance matrices and lag projections were obtained in each state/group (adult N0-N3, infants 6, 12, and 24 months) by computing each quantity at the individual subject level (averaging across temporally contiguous epochs) and then averaging.

### Principal components analysis (PCA)

Principal components analysis (PCA) decomposes FC matrices into spatio-temporal components. Each component represents a particular topography (the eigenvector) that accounts for a particular fraction of total variance. Thus, PCA generates a rank-ordered description of FC patterns. Here, we use PCA descriptively to compare the rank orders of the first few PCs across groups, e.g., adult N0 (wake) FC vs. 6 month-old FC.

### Statistical analyses

We also use PCA to assess statistical significance in group comparisons, e.g., 6-month old vs. adult N0 functional connectivity. PCA is applied to the group difference FC matrix and the resulting eigenvalues compared to thresholds derived by simulation of the null hypothesis (H_0_: no difference between groups) using permutation resampling. Thus, subjects pooled over both groups (e.g., 6-month old infants and N0 adults), are randomly assigned to surrogate groups (preserving true group sizes), PCA is computed, and the distribution of greatest eigenvalues is compiled over 1000 realizations of H_0_. Statistical significance then is assessed as the fraction of surrogate eigenvalues greater than the eigenvalue corresponding to the true group difference principal component.

## Results

### Functional connectivity

**Fig 1:**
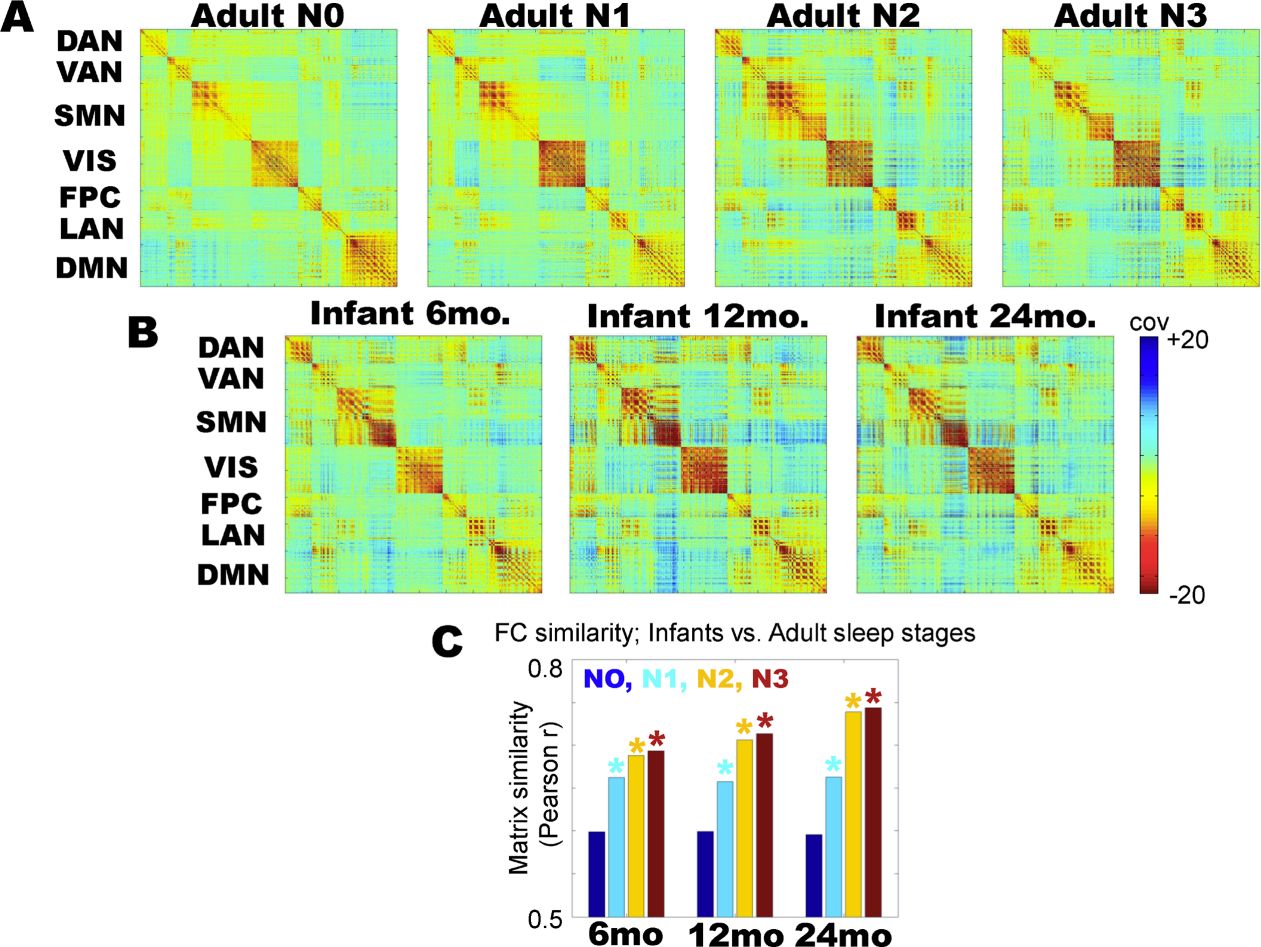
Zero-lag functional connectivity (covariance) matrices, sorted into cortical networks. (A) Functional connectivity in adults, in wake (N0) and in non-REM sleep (N1-N3). (B) Functional connectivity in 6, 12 and 24 month old children. (C) Functional connectivity (FC) similarity between each childhood matrix and adult wake/sleep stages (N0-N3). Similarity is computed by taking the correlation over all unique (matrix upper triangle) pairs of covariance values over the entire brain. At all ages, infant functional connectivity is more correlated with adult sleep (N1-N3) than adult wake (N0); * = p < 0.01 from permutation resampling on adult N0 vs. N1-N3 matrices with Bonferroni correction for multiple comparisons. Early childhood functional connectivity is most correlated with adult N3 sleep functional connectivity at all ages, although the difference between N2 and N3 sleep is not statistically significant.

We first examined functional connectivity in sleeping children and in adult wake/sleep. Fig 1 displays cortical functional connectivity matrices in adult wake (adult N0), adult non-REM sleep stages (adult N1-N3), as well as in childhood (6 months, 12 months, and 24 months). The matrices have dimensions voxels × voxels, where the voxels ((6mm)^3^ cubes) are sorted by resting state network affiliation (as in [10]); although various alternative network definitions could be applied, the specific choice of parcellation in Fig 1 is obviated by forthcoming whole-brain analyses. Visually comparing the adult vs. child FC matrices, it appears that the child matrices more closely resemble adult N2/N3 sleep as compared to adult N0 (wakefulness). To quantify this impression in a side-by-side comparison, we computed the correlation between all unique (matrix upper triangle) whole-brain FC values between children and adult N0-N3; the results are shown in Fig 1C. Statistical significance of the difference in 6–24 month old FC and adult N0 vs. N1-N3 FC was assessed using permutation resampling on adult wake sleep stages (e.g., 6 months:N0 vs. 6 months:N1, etc.) with Bonferroni correction for multiple comparisons. Functional connectivity in the asleep 6-, 12-, and 24-month old children is least like adult N0, and most like adult N3 sleep, although the difference between N2 and N3 is not statistically significant. These results (Fig 1C) demonstrate that sleeping childhood functional connectivity at all ages (6–24 months) is least correlated with adult wakefulness (N0) and most correlated with adult slow wave sleep (N3). Thus, the FC structure of sleeping children more closely resembles adult sleep than adult wakefulness.

**Fig 2:**
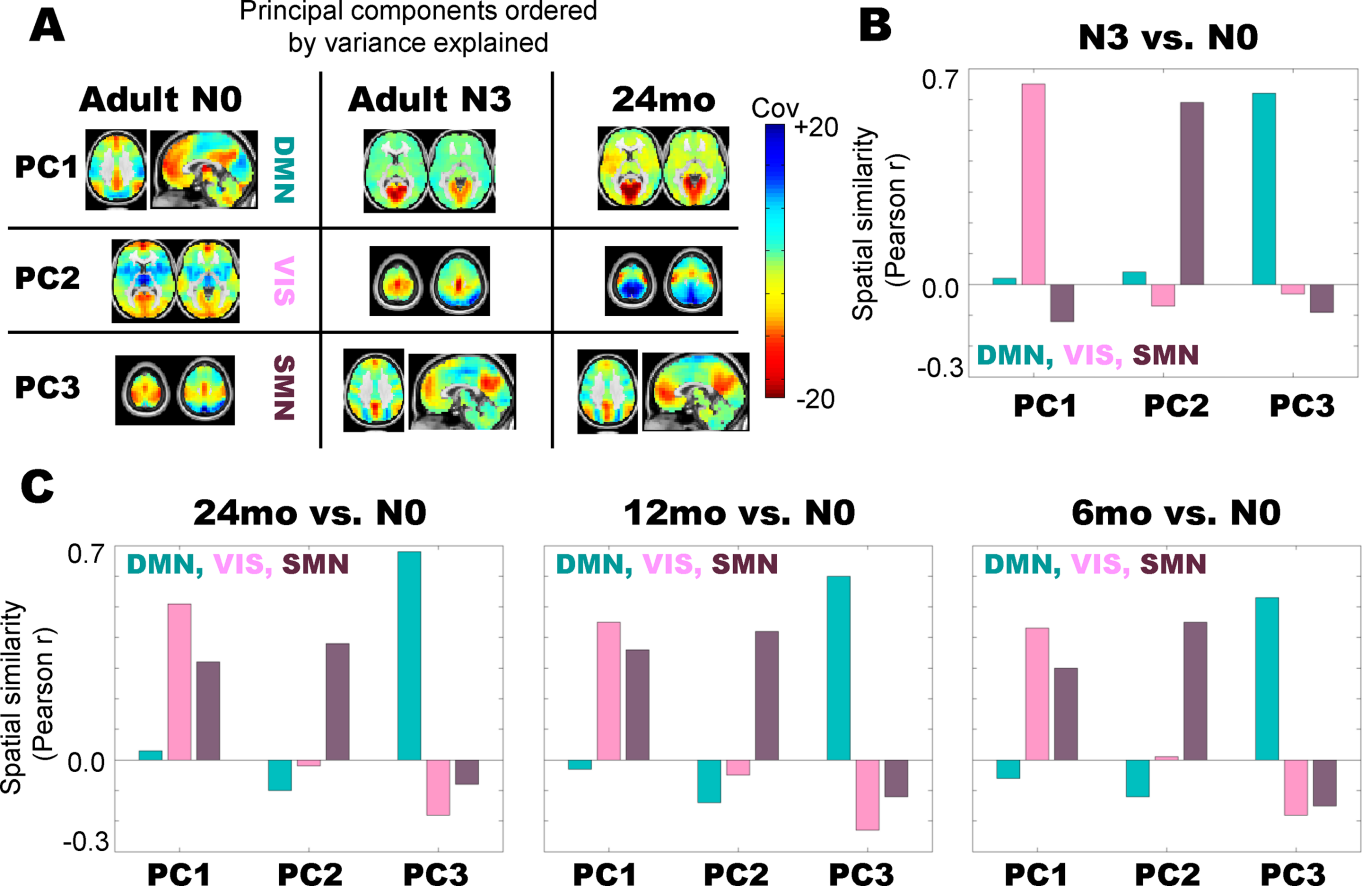
Principal component structure of adult N3 (slow wave) sleep best matches early childhood data. (A) First 3 principal components (PCs) in adult N0 (wake), adult N3 (slow wave sleep), and 24 month olds, ordered by variance explained. The topographies of the first 3 PCs in N0 (wake) adults reflect the default mode network (DMN), visual network (VIS), and somatomotor network (SMN), respectively. In contrast, it is visually evident that the component order in adult N3 (slow wave sleep) and 24 months old is visual network, somatosensory network, and default mode network, respectively. (B) Quantitative analysis of spatial correlations between the first 3 PCs in adult N3 vs. the first three components in adult N0 (labeled DMN, VIS, and SMN) demonstrates that the first adult N3 component most closely matches the N0 visual topography, the second adult N3 component most closely matches the N0 somatomotor topography, and the third adult N3 component most closely matches the N0 default mode network topography. (C) Quantitative analysis of spatial correlations between the first 3 PCs in data collected at 24, 12, and 6 months of age vs. adult N0 shows the same pattern of component re-ordering as in the adult N3 vs. adult N0 comparison.

To further understand the features which differentiate adult/childhood sleep from adult wakefulness, we analyzed the principal component structure of the functional connectivity in each condition. Principal components analysis (PCA) provides a rank-ordered description of variance in FC patterns. Applying PCA to adult N0 (wake) data, we find that the first PC, which accounts for the most variance in the data, corresponds qualitatively to a default mode network (DMN) topography (Fig 2A). The second and third PCs in adult wakefulness (N0) correspond qualitatively to visual (VIS) and somatosensory (SMN) topographies, respectively. To verify the qualitative assignment of network topography labels, we further compared the presently derived PCA topographies to a priori defined templates of DMN, VIS, and SMN topographies using spatial correlations and arrived at the same assignments. Thus, the most variance in adult waking rs-fMRI functional connectivity is driven by the default mode network topography, followed by visual and somatosensory topographies, respectively. These results are in line with recently published results in awake, young adults [37].

In contrast, Fig 2A shows that the first PC in adult slow wave sleep (N3) corresponds to a visual topography, followed by a somatosensory topography (PC2) and then a default mode network topography. Hence, in terms of variance accounted for, the relative contributions of these three networks have been reordered. Visual and somatomotor areas drive the most variance during adult slow wave sleep, as opposed to the default mode network topography, which drives the most variance in adult wakefulness. This finding recapitulates many prior results demonstrating lowered DMN functional connectivity in adult sleep vs. wakeful state [9, 12]. Critically, the principal component ordering in 24 month olds (Fig 2A) resembles adult sleep: PC1 is a visual topography, followed by the somatosensory network and then the default mode network.

We quantitatively assessed re-ordering of the rs-fMRI component structure by computing spatial correlations between PC’s derived across conditions. Fig 2B shows spatial correlations between PCs 1–3 in adult slow wave sleep (N3) and PCs 1–3 in adult wake (N0), where the adult N0 components are labeled DMN, VIS, and SMN, respectively. Note that PC1 in N3 sleep is most correlated with the visual component from wakefulness (N0 PC2). PC2 in N3 sleep is most related to the SMN component from wakefulness (N0 PC3), and PC3 in N3 sleep is most related to the DMN in adult wakefulness (N0 PC1). Hence, Fig 2B quantitatively confirms the impression of factor re-ordering evident in Fig 2A.

Fig 2C shows the spatial correlations between PC topographies in sleeping children and the DMN, VIS, SMN components from adult wakefulness. Note that in each case, PC1 in the children most closely matches the adult visual component, PC2 most closely matches the adult SMN component, and PC3 most closely matches the adult DMN component. These results demonstrate that the factor structure of the childhood data resembles that in adult slow wave sleep, and suggest that the observation of reduced functional connectivity in the early-age DMN compared to awake adults may be at least in part attributable to a wake vs. sleep effect. Eigenspectra for the PC topographies analyzed in Fig 2 are shown in S2 Fig, and demonstrate that the ordering of the first three principal components is well-separated in our rs-fMRI data.

### Propagation

We have recently demonstrated the existence of multiple, reproducible propagation patterns in the rs-fMRI BOLD signal [14, 15]. Moreover, we have found that, in healthy young adults, infra-slow propagation sequences are strongly altered as a function of wake vs. sleep [10, 18]. These results raise the question of whether the propagation structure of rs-fMRI in children more closely resembles adult wakefulness or adult sleep.

**Fig 3:**
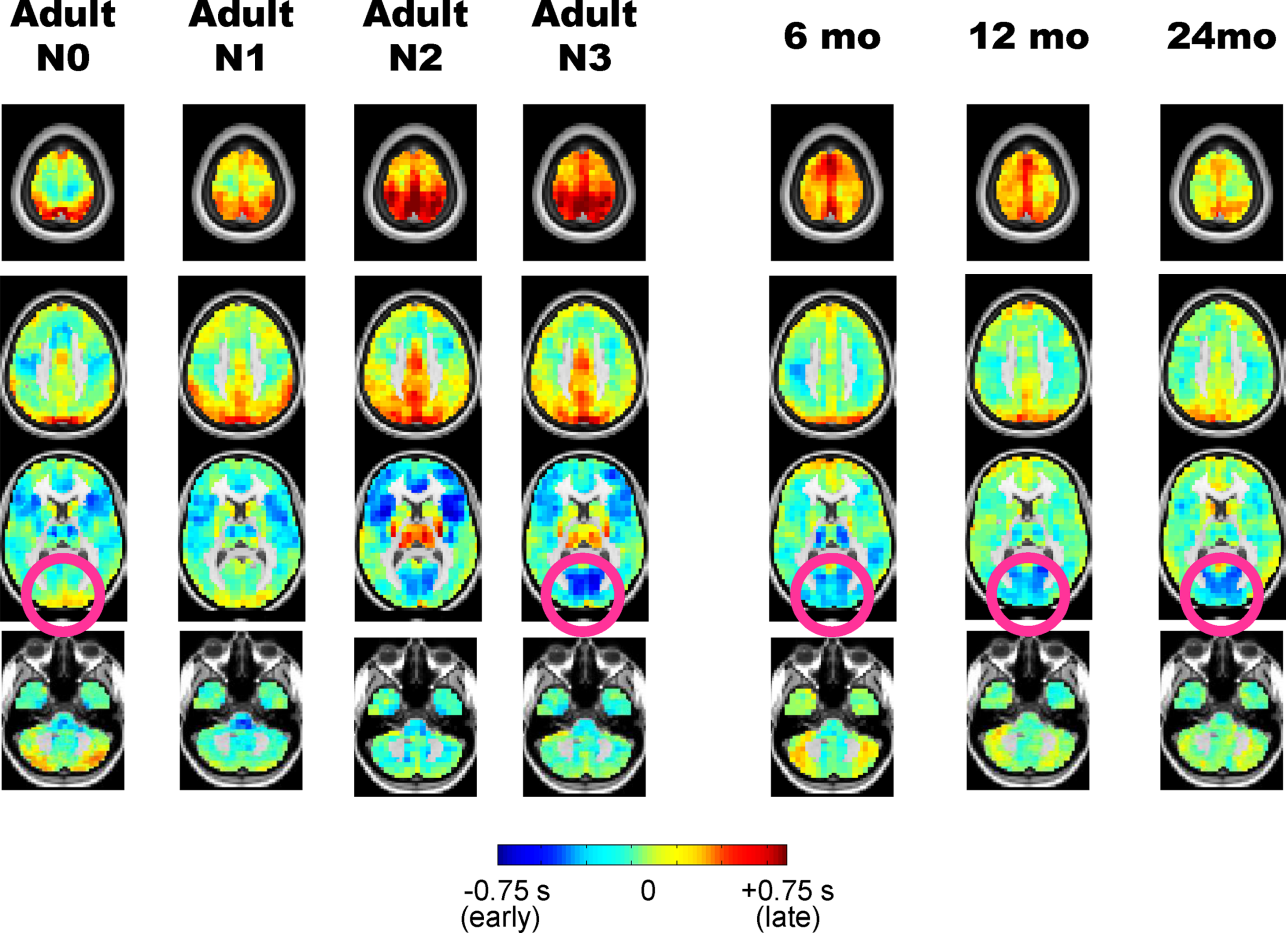
Propagation analysis of the resting-state fMRI BOLD signal in adult sleep and early childhood. Columns show temporal lag projection maps in adult wake/sleep stages as well as in children aged 6–24 months. Blue colors indicate regions where spontaneous BOLD signal activity tends to be early with respect to the rest of the brain; red colors indicate regions which tend to be late with respect to the rest of the brain. Pink circles contrast visual earliness in adult N3 sleep and infants against adult N0 wake.

Fig 3 illustrates lag projection maps (see Methods; [15]) in adult wake/sleep, as well as in children. Notice that the pink circles in adult N0 vs. adult N3 demonstrate that the visual cortex is much earlier (more “blue”) during sleep than wake, a fact which we have previously reported [10]. The pink circles in the pediatric lag projection maps qualitatively highlight that visual cortex earliness is also present at all ages but is closest to adult N3 at 24 months. There are also aspects of the childhood sleep lag projections that more closely match adult N0 than adult N3, for example, in medial motor areas illustrated in the 2nd row of Fig 3.

**Fig 4:**
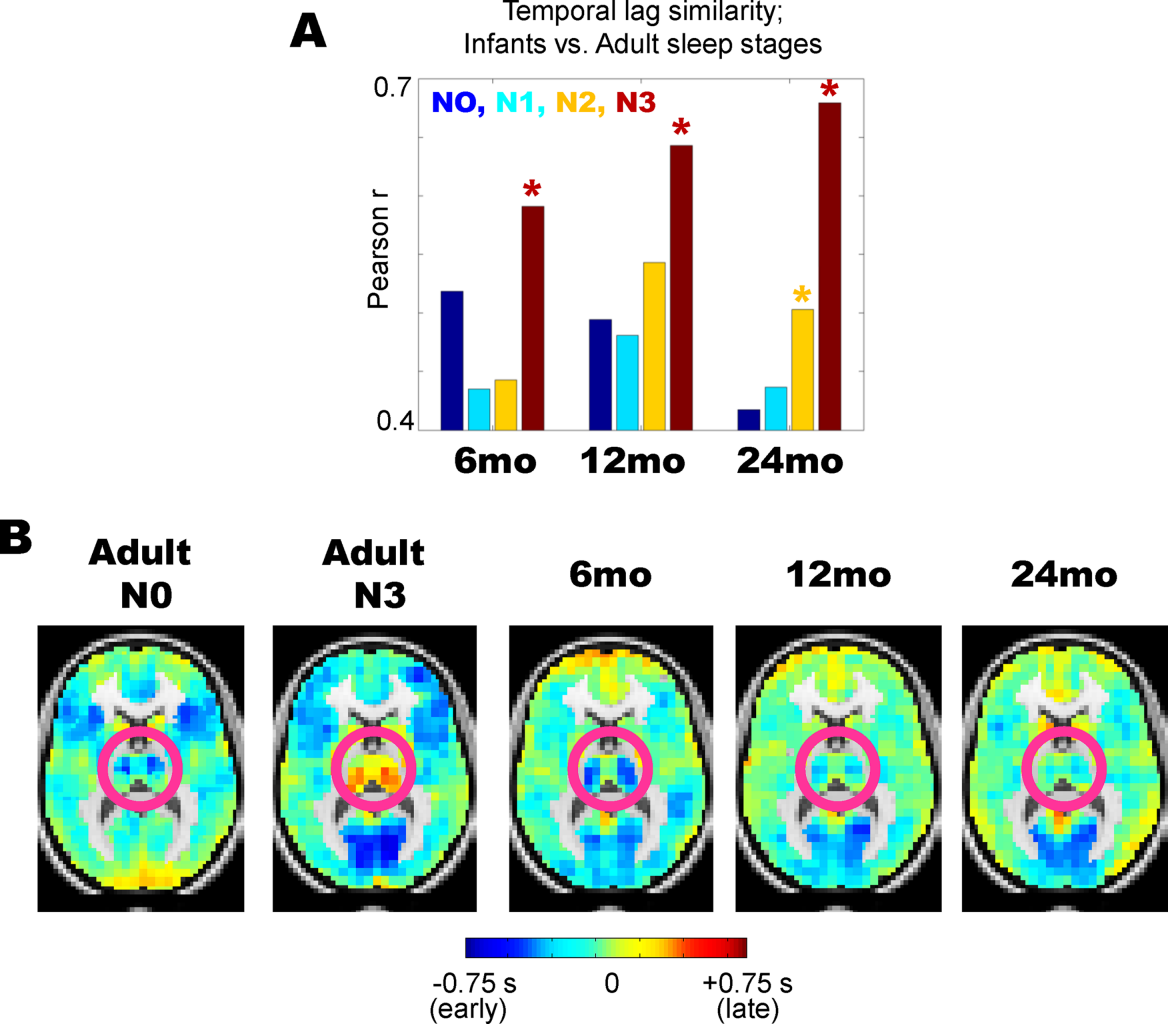
Propagation patterns in early childhood vs. adult wake/sleep. (A) Temporal lag projection maps at all ages (6–24 months) most closely match adult N3 sleep; * = p < 0.01 from permutation resampling on adult N0 vs. N3 lag projections with Bonferroni correction for multiple comparisons. (B) Despite the overall spatial correlation between early childhood lag projections and adult N3 sleep lag projections, there are critical differences in the thalamus (highlighted in pink circles). Thalamus is generally late with respect to the rest of the brain in adult N3 sleep. In contrast, in 6 month olds, the thalamus is early with respect to the rest of the brain, more akin to adult N0 wake. Note that this earliness fades with aging (e.g., the thalamus becomes less blue), indicating a possible development toward adult-like sleep features.

We quantitatively examined similarity in lag projections between sleeping children and adult wake/sleep by computing spatial correlations. The results, shown in Fig 4A, demonstrate that at each age, infants’ propagation patterns are more correlated with adult slow wave (N3) sleep than adult wakefulness. Part of the basis of this similarity is illustrated in Fig 4B. The pink circles highlight the thalamus and striatum; note that both of these structures are early (or blue) in adult N0 wake, but late (yellow/orange) in adult N3 sleep, as previously reported [10]. Although Fig 4A shows that in general childhood sleep propagation patterns resemble N3 sleep more than N0 wake in adults, note that the thalamus is early (blue), and hence adult wake-like in 6 month old infants (Fig 4B). Interestingly, the degree of earliness in the thalamus decreases across 6–24 months of age (Fig 4B), moving in the direction of the adult N3 sleep structure.

**Fig 5:**
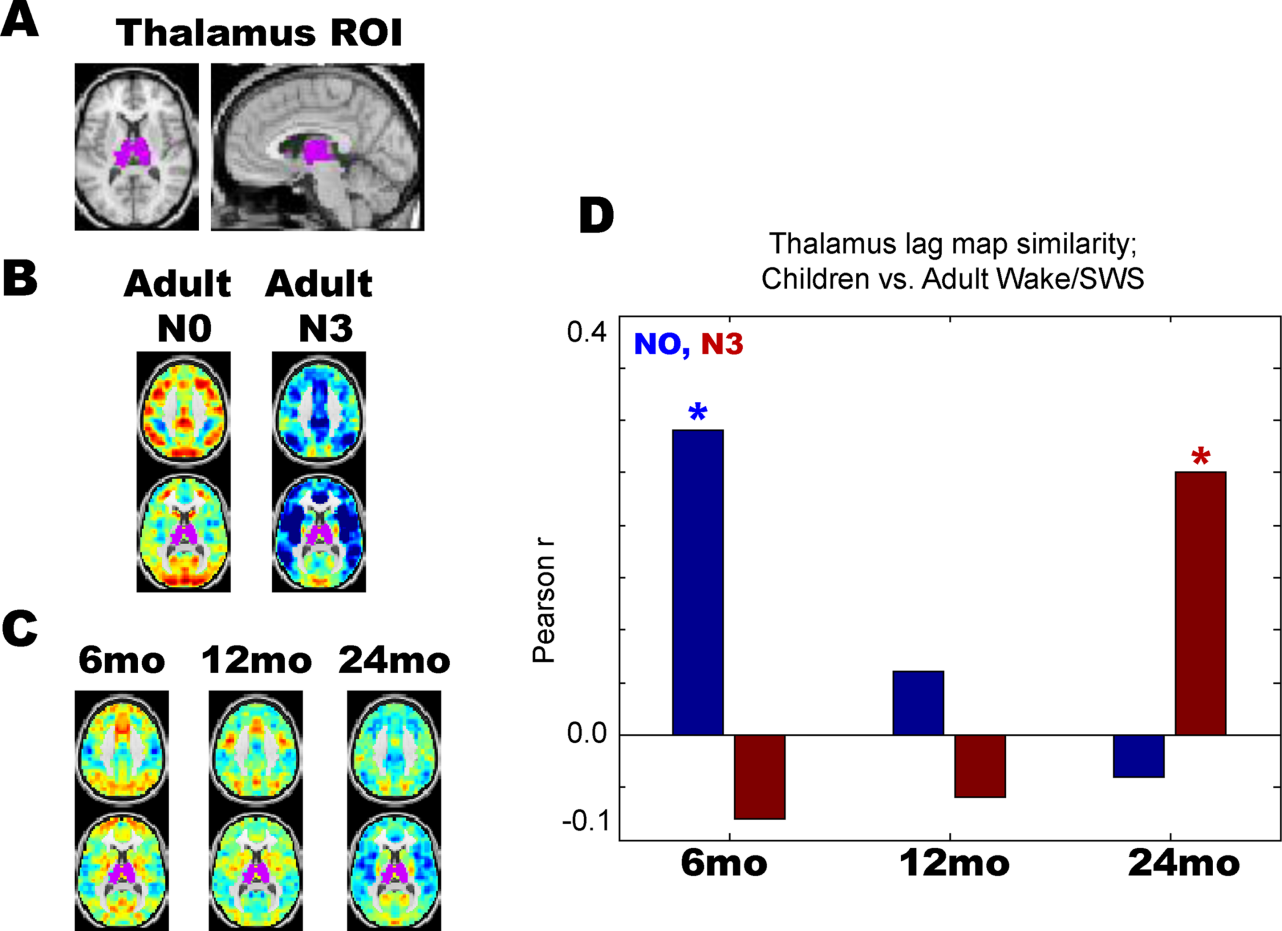
Thalamic lag structure in early childhood vs. adult wake/sleep. (A) Whole thalamus seed-region. (B) Thalamus-seeded lag maps in adult wake (N0) and slow wave sleep (N3). As previously published, cortex is generally late (red) with respect to thalamus during wake, but generally early (blue) with respect to thalamus during N3 sleep. (C) Thalamus-seeded lag maps in early childhood. The 6-month old lag map shows most of cortex is late with respect to thalamus, akin to adult wake. In contrast, the 24-month old lag map shows most of cortex is early with respect to thalamus, akin to adult N3 sleep. (D) Thalamic lag map in sleeping 6-month olds is significantly more correlated with adult wake than adult sleep, whereas the opposite is true for sleeping 24-month olds; * = p < 0.01 from permutation resampling on adult N0 vs. N3 lag projections with Bonferroni correction for multiple comparisons. No statistically significant difference was found at 24-months.

We examined the qualitative findings shown in Fig 4B by computing thalamus-seeded lag maps, which simply depict the temporal delay between each voxel and the average timecourse derived from the whole thalamus (Fig 5A). As previously reported, thalamic lag structure is markedly altered across adult wake (N0) and slow wave sleep (N3) [10]. Specifically, most of the cortex is red or “late” with respect to thalamus during adult wakefulness, whereas most of the cortex is blue or “early” with respect to thalamus during adult N3 sleep (Fig 5B). Thalamus-seeded lag maps computed in sleeping 6-, 12-, and 24-months reveal a clear progression (Fig 5C) such that the 6-month old thalamic lag map more closely resembles adult wake than adult sleep (more red than blue in cortex), whereas the 24-month old thalamic lag map more closely resembles adult sleep than adult wake (more blue than red in cortex). These results are quantified in Fig 5D, where the 6-, 12-, and 24-month thalamic lag maps are spatially correlated with adult wake/sleep thalamic lag maps.

## Discussion

### Summary of principal findings

To date, all resting state fMRI in infants and toddlers has been acquired during sleep, which gives rise to the interpretive problem of distinguishing between the effects of arousal state versus development. To investigate this question, we compared rs-fMRI acquired in 6-, 12-, and 24-month old children to adult data acquired during well-characterized states of arousal (N0, N1, N2, N3). Our primary result is that functional connectivity (i.e., zero-lag correlation) more closely resembles adult slow wave sleep (N3) than adult wake (N0) (Figs 1 and 2). Moreover, this result is most robust in 24-month toddlers as opposed to 6-month old infants. These findings caution against concluding that differences between sleeping infants and awake adults are solely attributable to development. In particular, we find that decreased functional connectivity in the default mode network, compared to visual and somatomotor networks, is a feature of both infant sleep and adult slow wave sleep. However, in contrast to the functional connectivity results, propagation patterns in 6 month old infants are more closely related to adult wake than adult N1/N2 sleep (p < 0.01 by permutation resampling). Thus, the effects of development on intrinsic brain activity partially dissociate depending on whether the measure is conventional functional connectivity (i.e., zero-lag correlation; Figs 1 and 2) vs. lag analysis (Figs 3–5). Implications of these observations are considered below.

### RS-fMRI correlates of the ontogeny of sleep

In infants, quiet sleep is distinguished from wake (stage N0) and active sleep (stage REM or N4) by the presence of slower electroencephalographic (EEG) activity [38]. However, sustained, slow (< 4 Hz) delta, i.e., the hallmark of the deepest stages of SWS in adults, does not appear until somewhat later, i.e., around 2–3 year of age [39]. By EEG features, REM resembles wakefulness at all ages, but this resemblance is especially pronounced in infants [40, 41]. Thus, the EEG features of wakefulness, REM, and SWS become progressively more differentiated as development proceeds.

We find a resting state fMRI correlate of this principle in the temporal lag results. Specifically, the temporal lag features of 6-month old sleeping infants are more adult wake-like than adult sleep-like (Figs 3–5). This effect fades with development such that the lag structure in 24-month old toddlers approaches that of N3 (SWS) in adults. However, even in the 24-month group, thalamic lag values are near zero (Fig 3), that is, no longer clearly early (~-0.6 sec in the 6-month old group) but neither is it clearly late (~+0.6 sec in awake adults). Active sleep accounts for about 50% of all sleep time in newborn infants but this fraction decreases by approximately 20% over ages 6–24 months [40]. Accordingly, it is very likely that the proportion of REM sleep in the 6-month old data is greater than in the 24-month old data. Thus, the wake-like thalamic propagation feature found in infant sleep (Fig 4B; Fig 5), especially in the 6 month-old group, could very well reflect REM-sleep, which is known to resemble wakefulness on EEG [41]. Moreover, PET studies in adults have shown that cerebral blood flow in the thalamus during REM sleep is comparable to wakefulness, but is sharply reduced in SWS. This observation provides a possible explanation for similarity of thalamic lag features of 6 month-old infants vs. awake adults [42]. Unfortunately, acquiring motion-free fMRI during REM sleep in adults is technically very difficult. Hence, direct comparisons between early childhood and adult REM sleep remains a challenge.

### Correspondence of FC with the broader developmental literature

In early brain maturation, primary cortices (e.g., sensorimotor, visual) develop before "higher order" areas in prefrontal and parietal regions. This developmental sequence is reflected in multiple disparate measures: metabolic activity [43], myelination of white matter [44], and imaging indices of gray matter maturation [45]. Thus, on the basis of this evidence, there is no question that human brain development follows a topographic sequence.

However, with respect to the resting state fMRI literature, the question is to what extent the aforementioned developmental changes are responsible for differences observed between sleeping children and awake adults. As several features of resting state functional connectivity follow the primary to higher order sequence, hypothesizing a link to developmental processes is entirely reasonable [7, 8, 46–48]. For example, in healthy term babies, functional connectivity within primary sensorimotor and visual RSNs is much better developed in comparison to higher-order RSNs, e.g., [2]. Moreover, functional connectivity within non-primary RSNs, e.g., the default mode and dorsal attention networks, becomes much better defined over the first two years of life, e.g., [1, 49].

Yet, our present data demonstrates that many of the same resting state functional connectivity effects along the primary/higher-order axis are found within adult populations contrasting wakefulness and slow wave sleep. This demonstrates that development is not required as an explanation for some sleeping child vs. awake adult differences. We therefore suggest that sleep itself, along with developmental processes, may be responsible for previously reported resting state functional connectivity differences between sleeping children and waking adults.

### Caveats and limitations

Limitations in of the present analysis include: (1) Cross-sectional imaging across ages, which provides an average view of changes with development, and (2) the present analysis compares adult vs. pediatric infant data acquired using different sequence parameters and slightly different preprocessing strategies. These technical factors likely contribute to some early childhood vs. adult differences in rs-fMRI, but the overall similarity between infant sleep and adult sleep argues that the major conclusions are unlikely attributable to these factors.

## Supporting information

S1 Fig Quality control metrics on infant data. Prior work has demonstrated that DVARS is an effective measure of frame-by-frame artifact in resting state fMRI which relates both to head motion artifact and other sources of spurious variance, such as respiration [50, 51]. Lower mean and standard deviation of DVARS values in an imaging run indicate greater immunity from artifact. Thus, to examine the quality of the infant data, all 483 imaging sessions (including scans at all ages and all diagnosis and familial risk categories) available in the IBIS database were plotted on the basis of mean DVARS vs. standard deviation (SD) of DVARS. The result demonstrates wide variability in DVARS measures (indicating the presence of artifact) in the infant resting state data. In the present study, we considered only data sets within one standard deviation (in mean DVARS and DVARS SD) of the lowest-artifact scans in the database (corresponding to the dots in the lower left of the image). This consideration led us to exclude scans with a mean DVARS > 7.5, or a DVARS SD > 5, leaving 337 out of the original 483 data sets. These 337 data sets were then restricted to only those corresponding to low risk negative subjects (not diagnosed with autism spectrum disorder (ASD) and did not have a sibling with diagnosed ASD), leaving 82 data sets. Finally, we considered only data sets with a minimum of 200 frames and at least 4 contiguous 60 second censoring-free epochs, as lags analysis is best applied to continuous stretches of fMRI data. This final consideration leads to the 45 data sets analyzed in this study.
(TIFF)

S2 Fig Eigenspectra for the principal components analysis in main text Fig 2. (A) Each correlation matrix principal component, analyzed in main text Fig 2, corresponds to an amount of variance accounted for in the data. These variance measures comprise an eigenspectrum, and the plot illustrates the eigenspectrum for each group analyzed in the present study, color-coded as described in the legend. We present separate plots for the adult and pediatric data simply for ease of viewing; the axes on both plots are identical. Visual inspection of the adult wake (blue) and adult sleep (pink) eigenspectra reveals that the amount of variance explained by the first three components in each condition is roughly equivalent. Thus, the change in the ranking of variances in the top three components, highlighted in main text Fig 2, is not accompanied by a dramatic change in the amount of variance explained by each component number. In other words, in the case of adult sleep, although the first component of the data has a default mode network topography during wakefulness, and a visual topography during slow wave (N3) sleep, the first component still accounts for ~20% of the variance in both conditions. A similar equivalence is found in the pediatric data. (B) For reference, the first three component topographies of adult N0, adult N3, and 24 month olds corresponding to the first three elements of the respective eigenspectra are reproduced from main text Fig 2.
(TIF)
