# Supplementary figures and images for: Resting-state fMRI in sleeping infants more closely resembles adult sleep than adult wakefulness
Source: PLoS One. 2017 Nov 17;12(11):e0188122. doi: 10.1371/journal.pone.0188122 (PMC5693436; doi:10.1371/journal.pone.0188122)

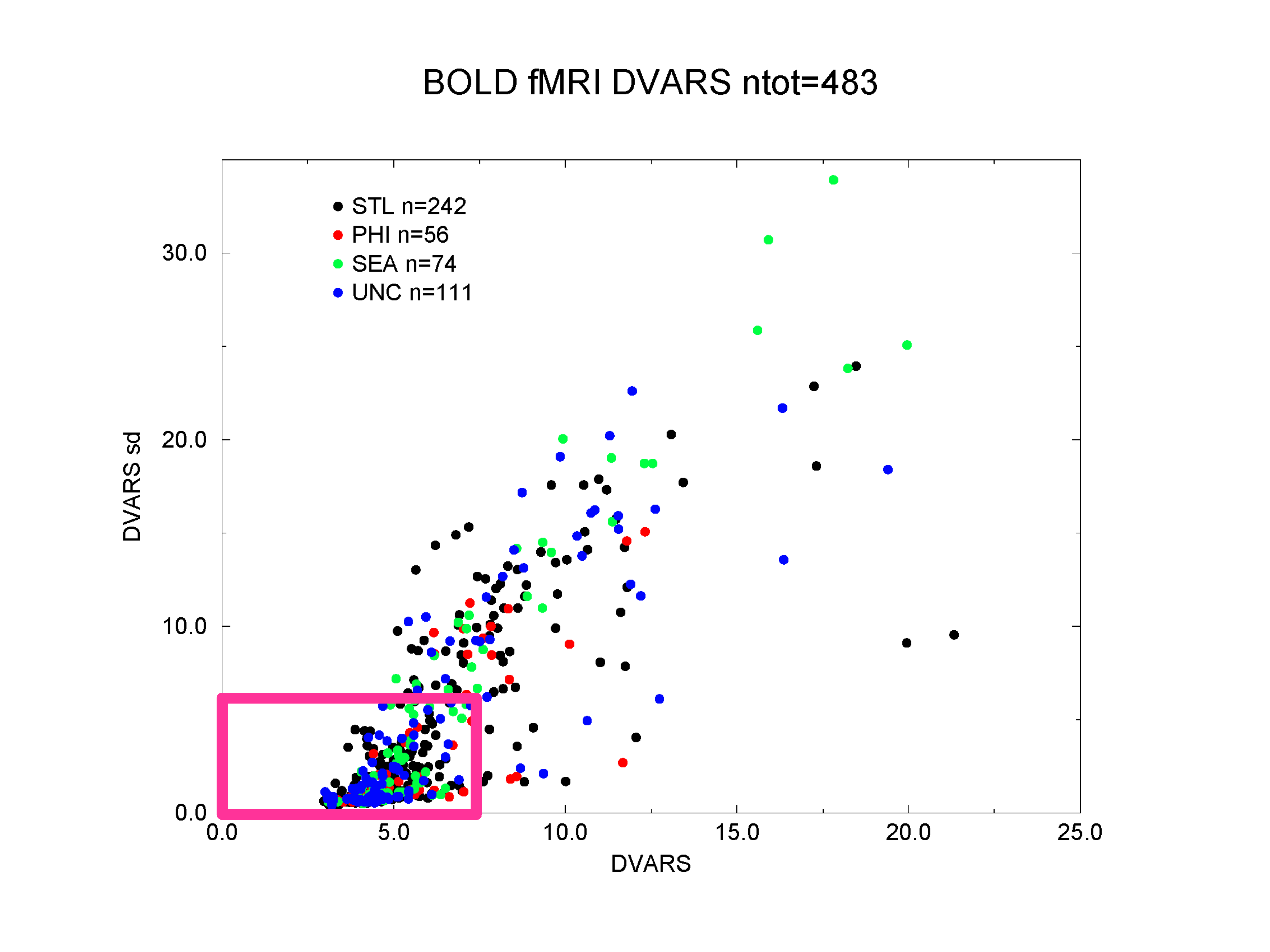

Supplement: S1 Fig — Prior work has demonstrated that DVARS is an effective measure of frame-by-frame artifact in resting state fMRI which relates both to head motion artifact and other sources of spurious variance, such as respiration [50, 51]. Lower mean and standard deviation of DVARS values in an imaging run indicate greater immunity from artifact. Thus, to examine the quality of the infant data, all 483 imaging sessions (including scans at all ages and all diagnosis and familial risk categories) available in the IBIS database were plotted on the basis of mean DVARS vs. standard deviation (SD) of DVARS. The result demonstrates wide variability in DVARS measures (indicating the presence of artifact) in the infant resting state data. In the present study, we considered only data sets within one standard deviation (in mean DVARS and DVARS SD) of the lowest-artifact scans in the database (corresponding to the dots in the lower left of the image). This consideration led us to exclude scans with a mean DVARS > 7.5, or a DVARS SD > 5, leaving 337 out of the original 483 data sets. These 337 data sets were then restricted to only those corresponding to low risk negative subjects (not diagnosed with autism spectrum disorder (ASD) and did not have a sibling with diagnosed ASD), leaving 82 data sets. Finally, we considered only data sets with a minimum of 200 frames and at least 4 contiguous 60 second censoring-free epochs, as lags analysis is best applied to continuous stretches of fMRI data. This final consideration leads to the 45 data sets analyzed in this study. (TIFF) [file pone.0188122.s001.tiff]

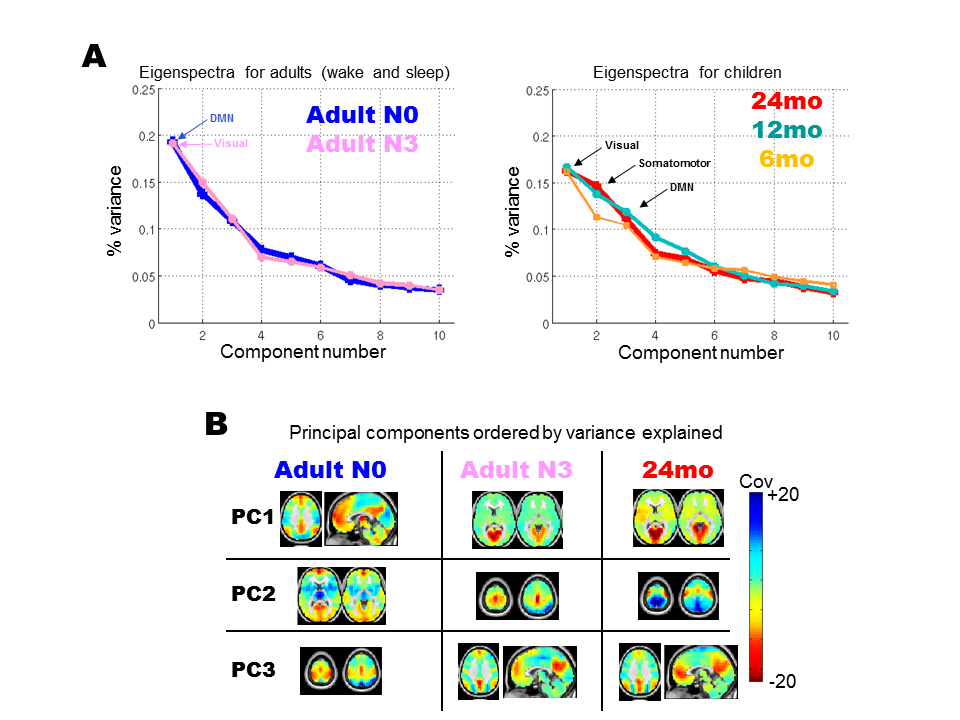

Supplement: S2 Fig — (A) Each correlation matrix principal component, analyzed in main text Fig 2, corresponds to an amount of variance accounted for in the data. These variance measures comprise an eigenspectrum, and the plot illustrates the eigenspectrum for each group analyzed in the present study, color-coded as described in the legend. We present separate plots for the adult and pediatric data simply for ease of viewing; the axes on both plots are identical. Visual inspection of the adult wake (blue) and adult sleep (pink) eigenspectra reveals that the amount of variance explained by the first three components in each condition is roughly equivalent. Thus, the change in the ranking of variances in the top three components, highlighted in main text Fig 2, is not accompanied by a dramatic change in the amount of variance explained by each component number. In other words, in the case of adult sleep, although the first component of the data has a default mode network topography during wakefulness, and a visual topography during slow wave (N3) sleep, the first component still accounts for ~20% of the variance in both conditions. A similar equivalence is found in the pediatric data. (B) For reference, the first three component topographies of adult N0, adult N3, and 24 month olds corresponding to the first three elements of the respective eigenspectra are reproduced from main text Fig 2. (TIF) [file pone.0188122.s002.tif]
